# Congenital heart disease frequency in children undergoing MDCT angiography; a 4-year tertiary care hospital experience from Kabul, Afghanistan

**DOI:** 10.1259/bjro.20180032

**Published:** 2019-07-09

**Authors:** Tariq Alam, Muhammad Khurram Munir, Hidayatullah Hamidi

**Affiliations:** 1 Saidu Medical College, Saidu Teaching Hospitals, Swat, Pakistan; 2 Southern Health NHS Foundation Trust, Southampton, UK; 3 French Medical Institute for Children, Kabul, Afghanistan

## Abstract

**Objectives:**

Echocardiography and cardiac angiography are two main imaging modalities used for evaluating congenital heart diseases (CHDs). Evaluation of CHDs is now possible with Multidetector CT (MDCT) angiography in Afghanistan. To the best of researchers' knowledge, no published data is available on frequency of CHDs among children undergoing chest MDCT angiography in Afghanistan; hence, this study is first of its nature to be conducted in this context. To describe the frequency of CHDs among children who underwent chest MDCT angiography in radiology department at French Medical Institute for Mothers and Children (FMIC) from April 2010 to July 2014.

**Methods & materials:**

A retrospective, cross-sectional descriptive study was conducted at radiology department FMIC in Kabul, Afghanistan. The study population consisted of all paediatric patients (aged 1 day–17 years) who underwent chest MDCT angiography at radiology department FMIC from April 2010 to July 2014. All examinations were performed in arterial phase by 128 slice Siemens scanner after intravenous administration of non-ionic water-soluble contrast material (Omnipaque 350) at a volume of 2 ml/Kg. CT setup included non-electrocardiogram gated CT, CT dose index 5–10 and dose–length product 120–200, with post-processing following initial scan. CT reports were reviewed from Radiology Information System. Data collection tool was developed and data were analysed using SPSS v. 22. Frequencies and proportion were calculated for various CHDs.

**Results:**

A total of 942 cases of contrast enhanced chest MDCT examinations were performed during this period. Out of these, 212 cases with CHDs were recruited, from which 29 cases were excluded because of undergoing previous surgical procedures or had incomplete CT reports. Remaining 183 cases (*n* = 183) of CHDs were included for further analysis. A total of 107 patients (58.5%) were male and 76 (41.5%) were female. The patients aged 1 day–17 years (mean age 4.47 + 4.76 standard deviation). A total of 87 patients (47.5%) had solitary anomalies while 96 patients (52.5%) had more than one defect. In terms of location, 20 cases (10.9%) were isolated intracardiac anomalies, 116 cases (63.4%) were isolated extracardiac anomalies and 47 cases (25.7%) had mixed intra- and extracardiac anomalies.

**Conclusion:**

Given the frequency, it is clear that CHDs is a complex health problem in Afghan paediatric population. MDCT angiography can be considered as a non-invasive, readily available diagnostic tool in evaluation of complex cardiac anomalies after initial evaluation.

**Advances in knowledge:**

MDCT evaluation of CHD as an alternative to echo/angiography has become more important in a country where there is severe shortage of interventional cardiologists.

## Introduction

Congenital heart diseases (CHDs) are defined as gross structural abnormality of the heart and or intrathoracic great vessels that are actually or potentially of functional significance.^1^ CHDs still remain a major cardiac problem in children.^2^

Cardiac imaging plays an important role in establishing the diagnosis, management and follow up after corrective or palliative surgery.

Echocardiography and cardiac angiography are two main imaging modalities used for evaluating CHDs.

Although echocardiography remains the first-line non-invasive imaging modality for evaluation of CHDs,^2^ but its inherent limitations, *e.g*. limited acoustic window, poor spatial resolution [when compared with multidetector CT (MDCT)] and operator dependency restricts its utility. These limitations become more pronounced in cases with post-surgical sternal wires and mediastinal scar tissue. Lastly, echo may not be sufficient when evaluating extracardiac lesions and/or vessels.^3^

Conventional angiography; the formerly accepted gold-standard for detection of CHDs is now uncommonly performed for diagnostic reasons alone.^4^ Its main limitations are two-dimensional nature, relatively invasive approach, need for larger volume of intravascular contrast material, frequent need for general anaesthesia and greater radiation dose compared to CT.^4,5^ Also, specialist availability, especially interventional cardiologist also needs to be accounted for in a country like Afghanistan.

CT and MRI can be used as non-invasive modalities, especially in the evaluation of pre- and post-operative cardiovascular anatomy.^6^ Cardiac CT examination can accurately depict intra- and extracardiac anomalies.^6^ Newer CT scanners yield images with better temporal and spatial resolution, greater anatomic coverage per rotation and more consistent enhancement with a lesser volume of intravascular contrast material, and higher-quality two-dimensional reformation and three-dimensional reconstruction owing to acquisition of an isotropic data set.^7^

Evaluation of CHDs is now possible with MDCT angiography in Afghanistan. To the best of researchers’ knowledge, no published data is available on frequency of congenital heart diseases among children undergone chest MDCT angiography in Afghanistan; hence, this study is being the first of its nature conducted in this context. Information generated from this study is likely to pave the way for further researches in Afghanistan in this field; as well as to promote awareness regarding the rule of MDCT angiography in evaluation of CHDs and frequency of various CHDs among health care professionals and general public.

### Objectives

To describe the frequency of congenital heart diseases among children who underwent chest MDCT angiography at radiology department of French Medical Institute for Mothers and Children (FMIC) from April 2010 to July 2014

## Methods and materials

A retrospective, cross-sectional descriptive study was conducted at radiology department of FMIC. The study population consisted of all paediatric patients (aged 1 day to –17 years) referred from cardiologist/clinicians who showed signs/symptoms of congenital heart diseases underwent chest MDCT angiography at radiology department of FMIC from April 2010 to July 2014.

All examinations were performed by 128 slice single source Siemens SOMATOM Definition AS scanner with gantry rotation time of 0.33 s. Collimation was made from the thoracic inlet to level of cardiac apex. Reconstruction of smooth, Kernel of 20–30 was performed in mediastinal window. Scans were done in arterial phase after intravenous administration of non-ionic water-soluble contrast material (Omnipaque 350) at a volume of 2 ml/Kg. CT set-up included non-electrocardiogram (ECG) gated CT, CT dose index 5–10 and dose–length product 120–200, with post-processing including axial MPR and virtual rendered technique following initial scan. Bolus tracking was performed. Region of interest was selected aortic was and the scan was stared when the Hounsfiedl unitHU reached 200. In majority of the cases sedation was not need. A smaller number of cases were given rectal Midazolam (1 mg/Kg).

CT reports were reviewed from Radiology Information System (RIS) by certified radiologist with 5 years of experience. The patients were referred by clinicians of different specialties for various reasons (cardiovascular, respiratory, traumatic and neoplastic processes) from over the country. Data collecting tool was developed and data were analyzed using SPSS v. 22.

Frequencies and proportion were calculated for various CHDs.

### Inclusion criteria

All paediatric patients (aged 1 day– to 17 years) who underwent chest MDCT angiography at radiology department of FMIC from April 2010 to July 2014 following referral from cardiology/allied clinicians.

### Exclusion criteria

Patients with known CHDs who had undergone any treatment procedure and patients with incomplete CT reports

## Results

In total, 212 cases with CHDs were recruited. Out of these, 29 cases were excluded as patients had undergone previous surgical procedures or did not have complete CT report. Remaining 183 case of CHDs were included for further analysis. Male to female ratio was 107 (58.5%) and 76 (41.5%) respectively. Patients age ranged from 1 day to 17 years (mean age 4.47 + 4.76 SD).

Out of this sample, 87 patients (47.5%) had solitary anomalies while 96 patients (52.5%) had more than one defect. About 20 cases (10.9%) were isolated intracardiac anomalies, 116 cases (63.4%) had isolated extracardiac anomalies and 47 cases (25.7%) had mixed intra- and extracardiac anomalies (Table 1).

**Table 1.** General characteristics (*n* = 183)

Characteristics	Frequency	Percentage (%)
Age (in years)		
Mean + SD	4.47 + 4.76	
Gender		
Male	107	58.5
Female	76	41.5
Number of defects		
More than 1	96	52.5
Solitary	87	47.5
Location of defects		
Isolated extracardiac defect	116	63.4
Mixed intra- and extracardiac	47	25.7
Isolated intracardiac defect	20	10.9
Part affected anomalies^a^		
Aorta	80	
RVOO and PTA	46	
Abnormal aortopulmonary connection	71	
APVC	20	
LPSVC	19	
Situs anomalies		
Situs solitus	169	92.3
Dextrocardia	8	4.4
Situs inversus	6	3.3

APVC, anomalous pulmonary venous connection; LPSVC, left persistent superior vena cava; PTA, pulmonary trunk atresia; RVOO, right ventricular outflow tract.

a Percentages were not calculated in “part affected anomalies” as some case(s), who had more than one anomaly, have been counted twice.

Situs anomalies: (Table 1 ﻿) A total of 169 cases (92.3%) were situs solitus, 8 cases (4.4%) being dextrocardia (DC) and 6 cases (3.3%) of situs inversus

Intracardiac defects: (Table 2﻿﻿﻿﻿) From 67 cases with intracardiac defects, ventricular septal defect (VSD) was the commonest lesion detected in 49 cases (73.1%) followed by atrial septal defect (ASD) seen in 18 cases (26.9%). One case was reported as single atrium and one case of conjoined twins with complex intracardiac connections.

VSD: isolated VSD was detected only in four cases, while VSD as part of Tetralogy of Fallot (ToF) was seen in 32 cases, VSD combined with pulmonary trunk atresia (PTA) in five cases, VSD with total anomalous pulmonary venous connection (TAPVC) in two cases, VSD with CoA in two cases (one of which was VSD with CoA+ASD), VSD with interrupted aortic arch (IAA) + ASD in one case, VSD with ASD in one case, VSD with patent ductus arteriosus (PDA): one case, and finally VSD with right-sided aortic arch (RtAA) in one case.

**Table 2.** Intracardiac [isolated (20)+Mixed (47)] lesions (*n* = 67)

Characteristics	Frequency	Percentage (%)
VSD	49^*a*^	73.1
Part of ToF +PTA Isolated +TAPVC +CoA (one case+ASD) +IAA+ASD +ASD only +PDA +RtAA	32 5 4 2 2 1 1 1 1	47.6 7.5 6.0 3.0 3.0 1.5 1.5 1.5 1.5
ASD	18^*a*^	26.9
+TAPVR Isolated +VSD^*a*^ +LPSVC	11 3 3 1	16.4 4.5 4.5 1.5
Single atrium	1	1.5
Conjoined twins with complex intracardiac lesions^*a*^	1	1.5

ASD, atrial septal defect; IAA, interrupted aortic arch; PDA, patent ductus arteriosus; PTA, pulmonary trunk atresia; RtAA, right-sided aortic arch; TAPVC, total anomalous pulmonary venous connection; ToF, tetralogy of fallot; VSD, ventricular septal defect.

a 3 cases with both ASD and VSD are counted twice.

**Figure 1.**
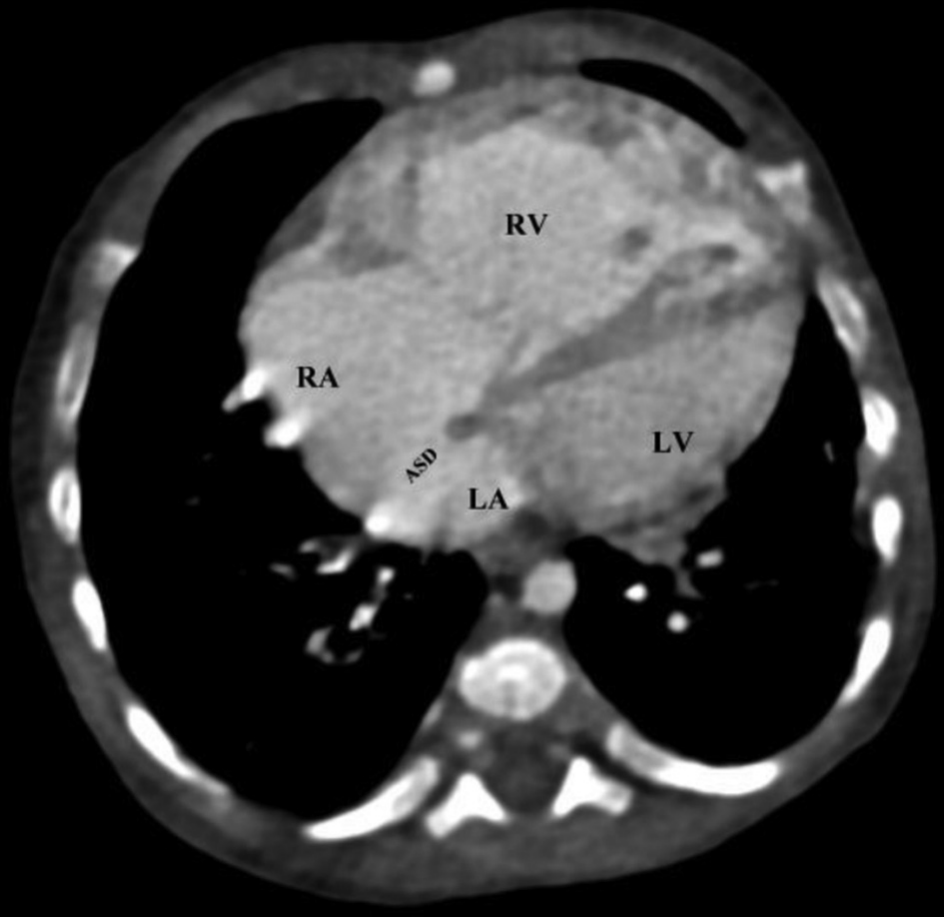
Figure 3 which subsequently drains to the LBCV and to SVC. The axial image through the cardiac chambers show a large ASD between the RA and LA facilitating a right to left shunt and making the condition compatible with life. ASD, atrial septal defect; LBCV, left brachiocephalic vein; LA, light atrium; RA, rightatrium; SVC, superior vena cava.

ASD: from the total 18 cases of ASD only three cases were isolated entities while 11 cases were associated with total anomalous pulmonary venous connection (TAPVC), three cases with VSD and one case with LPSVC (Figure 1) (Table 2﻿).

### Aortic anomalies (AA): (Table 3)

A total of 80 case were diagnosed as AA; 58 cases had anomalies in calibre [CoA, IAA and PCoA) while 19 cases had anomalies in orientation (RtAA), one case both in calibre and orientation (RtAA+CoA) and one case being anomalous in origin (Truncus arteriosus). Coarctation of Aorta (CoA): the most common congenital defect was CoA (35 cases). Out of these, 21 cases of CoA were isolated anomalies while 14 cases were associated with other intra or extracardiac anomalies (Table 3).

**Table 3.** Aortic anomalies (*n* = 80)

Characteristics	Frequency	Percentage (%)
CoA	35	43.8
Isolated +PDA +LPSVC +RtAA+ToF +VSD+ PDA+LPSVC +VSD+ASD +DC	21 9 1 1 1 1 1	26.3 11.3 1.3 1.3 1.3 1.3 1.3
IAA	12	15.0
+PDA Isolated +LPSVC +ASD+VSD + PDA	6 4 1 1	7.5 5.0 1.3 1.3
PCoA	11	13.8
+PDA Isolated +LPSVC +DC+LPSVC +ToF	6 2 1 1 1	7.5 2.5 1.3 1.3 1.3
RtAA	20^a^	25.0
+ToF Isolated +LPSVC +ToF+ALSCA +LPSVC+PAPVR + SI +SI+ToF +VSD +CoA	9 3 2 1 1 1 1 1^a^	11.3 3.8 2.5 1.3 1.3 1.3 1.3 1.3
Hypoplastic aortic root	1	1.3
hypo plastic aortic arch+PDA	1	1.3
Truncus arteriosus	1	1.3

ALSCA, Aberrant left subclavian artery ; ASD, atrial septal defect; IAA, interrupted aortic arch; PAPVR, partial anomalous pulmonary venous return; PDA, patent ductus arteriosus; PTA, pulmonary trunk atresia; RtAA, right-sided aortic arch; TAPVC, total anomalous pulmonary venous connection; ToF, tetralogy of fallot; VSD, ventricular septal defect.

a The case of RtAA associated with CoA is counted twice.

**Figure 2.**
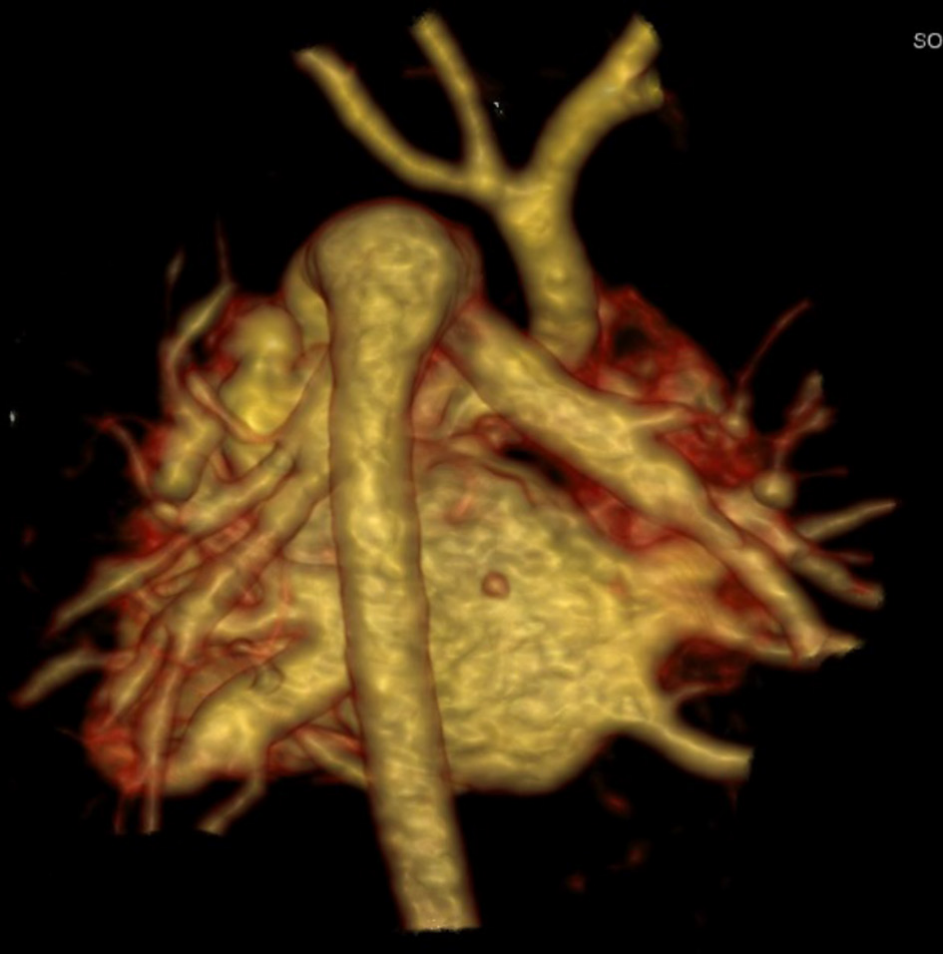
A 1-month-old tachypnic child: VRT reconstructed image from the posterior aspect of the heart shows interrupted aortic arch just distal to the origin of left subclavian artery (Type A). The descending aorta is getting its whole blood supply from the pulmonary trunk via large PDA. PDA, patent ductus arteriosus; VRT, virtual renderedtechnique.

IAA: interrupted aortic arch was the second most frequent pathology of aorta with 12 cases (Figure 2). Isolated IAA was seen in four cases while eight cases of IAA were combined with other intra- or extracardiac anomalies (Table 3). Pseudocoarctation of Aorta (PCoA): the next frequent anomaly of aortic arch was pseudocoarctation with a frequency of 11 cases. Isolated PCoA was seen in two cases while remaining nine cases were associated with other intra- or extracardiac anomalies (Table 3).

Hypoplastic aortic root was detected in one case and hypoplastic aortic arch also in one case.

### RtAA

From total 20 cases of anomalously oriented aortic arch; three cases were isolated entities while the remaining 17 cases were combined with other intra or extra cardiac anomalies (Table 3).

#### Right ventricular outflow tract (RVOO) and pulmonary arteries anomalies (PTA): (Table 4)

**Table 4.** Right ventricular outflow tract obstruction and pulmonary arteries anomalies (*n* = 46)

Characteristics	Frequency	Percentage (%)
ToF	32	69.6
+RtAA	12	26.1
ToF+RtAA only	9	19.6
+CoA	1	2.2
+ALSCA	1	2.2
+SI	1	2.2
Isolated +HPT +LPSVC +PCoA +DC +PDA +LPSVC+PAPVR + SI	10 4 2 1 1 1 1	21.7 8.7 4.3 2.2 2.2 2.2 2.2
Pulmonary arteries anomalies	18^a^	39.1
PTA	8^b^	17.4
+VSD	6	13.0
PTA + VSD only	4	8.7
+PAPVR	1	2.2
+DC	1	2.2
With IVS	2	4.3
HPT	6^a^ *^/^* ^b^	13.0
Isolated	2	4.3
+Tof	4	8.7
+Tof only	2	4.3
+DC	1	2.2
+Rt and Lt PA hypolasia	1	2.2
	
UPAA	2	4.3
AOUPA	2	4.3
Left side+RtAA	1	2.2
Isolated right-sided	1	2.2

ALSCA, Aberrant left subclavian artery ; AOUPA, anomalous origin of unilateral pulmonary artery; DC, dextrocardia; HPT, hypoplastic pulmonary trunk; IVS, interventricular septum; LPSVC, left persistent superior vena cava; MAPCA, major aortopulmonary collateral artery; PAPVR, partial anomalous pulmonary venous return; PCoA, pseudocoarctation of aorta; PDA, patent ductus arteriosus; RtAA, right-sided aortic arch; SI, situs inversus; ToF, tetralogy of fallot; UPAA, unilateral pulmonary artery atresia; VSD, ventricular septal defect.

a The four cases shared with ToF (all being HPT) are counted repeatedly.

b Four cases had associated MAPCAs.

A total of 46 patients had right ventricular outflow tract and pulmonary artery anomalies. About 32 cases (69.6%) were ToF with associated anomalies, while 14 cases (30.4%) were pathologies confined to pulmonary arteries only (Table 4).

**Figure 3.**
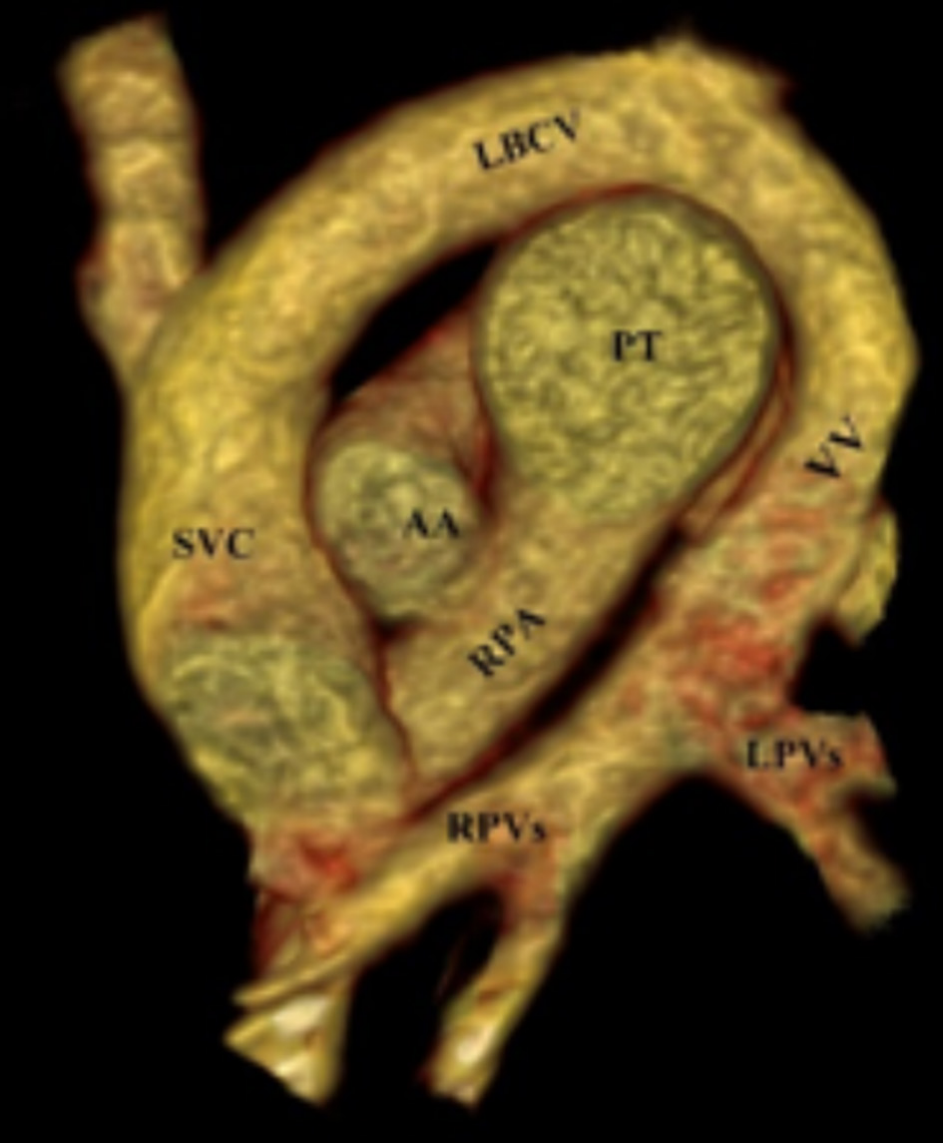
A 1-year-old cyanotic child. VRT reconstructed annotated coronal image through the great vessels show; right (RPVs) and left (LPVs) pulmonary veins join together making a vertical vein (VV). LBCV, left brachiocephalic vein ; LPV, left portal vein; RPV, right portal vein; SVC, superior vena cava; VRT, virtual rendered technique; VV, vertical vein.

ToF: from total 32 cases of ToF, 10 cases were isolated ToF while 22 cases had other simultaneous defects: 12 cases were associated with RTAA, 4 cases were associated with hypoplastic pulmonary trunk (HPT), 2 cases with left persistent superior vena cava (LPSVC), 1 case with pseudocoarctation of aorta (PCoA), 1 case with DC, 1 case with PDA and 1 case with LPSVC, partial anomalous pulmonary venous return (PAPVR) and situs inversus (SI). Pulmonary artery (PA) anomalies: a total of 18 patients had anomalies in PA (Figure 3). Pulmonary trunk atresia (PTA) was detected in eight cases, hypoplastic pulmonary trunk (HPT) in six cases, unilateral pulmonary artery atresia (UPAA) in two cases and anomalous origin of unilateral pulmonary artery (AOUPA) in two cases. PTA with VSD was seen in six cases. Out of six cases of PTA + VSD, one case was associated with PAPVR and one case with DC. In this PTA subgroup of eight cases, four cases were associated with major aortopulmonary collateral arteries (MAPCAs). PTA with intact interventricular septumIVS was seen in two cases.

### Hypoplastic Pulmonary Trunk (HPT)

From total six cases of HPT, four cases were associated with ToF, one case with DC, one case with associated hypoplasia of right and left pulmonary arteries. four cases in HPT group had associated MAPCAs. UPAA was seen in two cases, in both being right sided. Anomalous origin of unilateral pulmonary artery (AOUPA) was left sided in one case, associated with right sided aortic arch and MAPCA while the second case was isolated right side-AOUPA (Table 4).

### Abnormal aortopulmonary communications

(Table 5) a total of 71 patients had abnormal aorto-pulmonary connection. Patent ductus arteriosus (PDA) was the commonest type of abnormal aorto-pulmonary connection with 56 cases, followed by MAPCA 13 cases and AOUPA with 2 cases (Table 5). PDA: out of total 56 PDA cases, half of the cases (28), it was seen as isolated CHD, while in second half, PDAs were associated with other anomalies as described in Table 5. MAPCAs: total 13 cases of MAPCAs were seen. 7 cases combined with ToF, 5 cases combined with PTA and in 1 case was seen with HPT.

**Table 5.** Abnormal aortopulmonary connection (*n* = 71)

Characteristics	Frequency	Percentage (%)
PDA	56	78.9
Isolated +CoA +IAA +PCoA +LPSVC +VSD +ToF +HAA	28 10 7 6 2 1 1 1	39.4 14.1 9.9 8.5 2.8 1.4 1.4 1.4
MAPCA	13	18.3
+ToF +PTA +HPT	7 5 1	9.9 7.0 1.4
AOUPA	2	2.8
Total	71	100

MAPCA, major aortopulmonary collateral artery; PA, Pulmonary artery; PAPVR, Partial Anomalous Pulmonary Venous Return; PCoA, Pseudo Coarctation of Aorta; RVOT, Right ventricular outflow tract; SI, Situs Inversus; TAPVC, Total anomalous Pulmonary Venous Connection; UPAA, Unilateral pulmonary artery atresia.

### Anomalous pulmonary venous connection (APVC) (Table 6)

**Table 6.** APVC (*n* = 20) – Total *vs* Partial

Characteristics	Frequency	Percentage (%)
TAPVC	13	65.0
Supracardiac Infracardiac Cardiac +ASD +VSD	10 2 1 11 2	50.0 10.0 5.0 55.0 10.0
PAPVC	7	35.0
Isolated +ToF+SI +PTA	5 1 1	25.0 5.0 5.0
Total	20	100

AA, Aortic Anomalies; AOUPA, Anomalous origin of unilateral pulmonary artery; APVC, Anomalous Pulmonary Venous Connection; CHD, Congenital Heart Disease; HPT, hypo plastic pulmonary trunk; IVS, Interventricular septum; LPSVC, Left Persistent Superior Vena Cava; MAPCA, Major aorto-pulmonary collateral arteries; MDCT, Multi Detector Computed Tomography; PA, Pulmonary arteries; PTA, Pulmonary Trunk Atresia.

A total of 20 cases had APVC, 13 cases of TAPVC and 7 cases of PAPVC. The TAPVC was supracardiac in majority of cases (*n* = 10) while it was infracardiac in two cases and cardiac in one case. TAPVC was associated with ASD in 11 cases and with VSD in 2 cases.

PAPVR was an isolated entity in five cases while it was associated with PTA in one case and with ToF+SI in another case (Table 6).

### LPSVC

a total of 19 cases of LPSVC were detected. 3 cases were isolated while the rest of 16 cases were associated with other abnormalities (Table 7).

**Table 7.** LPSVC (*n* = 19)

Characteristics	Frequency	Percentage (%)
Isolated +PDA +ASD +ToF +DC +RtAA +PCoA +ToF+PAPVR + SI +CoA +CoA+VSD + PDA +IAA	3 2 2 2 2 2 2 1 1 1 1	15.8 10.5 10.5 10.5 10.5 10.5 10.5 5.3 5.3 5.3 5.3
Total	19	100

ASD, Atrial Septal Defect; CoA, Coarctation of Aorta; DC, Dextrocardia; IAA, Interrupted Aortic Arch; LPVSC, left persistent superior vena cava; PDA, Patent Ductus Arteriosus; RtAA, Right sided Aortic Arch; ToF, Tetralogy of Fallot; VSD, Ventricular Septal Defect.

## Discussion

The study demonstrates that various CHDs are fairly prevalent among Afghan paediatric population. More than half of the patients had more than one congenital heart defect(s). Aortic anomalies were the most common entities. As echocardiography is poor in evaluation of aorta, therefore patients with suspected aortic anomalies usually underwent CT. It is however, important to point out that cardiac MRI is not available in Afghanistan at the time of this study was undertaken, which leaves MDCT as the only cross-sectional modality available to investigate congenital lesions in this cohort.

CoA was the commonest aortic anomaly in our cases which is reported to account 5–7% of all CHDs.^8^ MZ Al-Azzazy reported that MDCT angiography with multiplanar and three dimensional techniques can be considered the modality of choice for pre-operative assessment of paediatric aortic coarctation.^9^ CoA with RtAA reported to be an exceedingly rare congenital anomaly^10^ was seen in one of our cases which was also associated with ToF.

Intracardiac lesion however reported the commonest CHDs, were less frequently detected in our study. This could be explained on the basis that ECG has the sensitivity of 100% in detection of intracardiac lesions compared to 85% sensitivity of non-ECG getting CT,^9^ therefore patients with isolated intracardiac defects without high suspicious for extracardiac defects should not routinely undergo CT. However, when planning for surgery, MDCT is useful for detection of other associated defects with for example, a single VSD, as an alternative to MRI. Also, in contrast to MRI, MDCT affords advantages, such as less scan time, minimal to no sedation, extremely useful in scanning of very small critically ill infants.

Right-sided aortic arch which is reported to occur in <0.1% of the population^11^ was detected in 20 of our patients. As the mirror type of RtAA is commonly associated with cyanotic heart disease,^12^ it was associated with ToF in almost half of the cases (nine cases) (Table 3).

ToF that consists of 7–10% of all CHDs^13^ was present of 32 of our cases. RtAA that is reported to be present in one-quarter of patients with ToF^13^ was present in more than one-quarter of our patients; 12 out of 32 (Table 4).

AOUPA branch from the ascending aorta accounts for only 0.12% of all CHDs.^14^ Right aortic arch is reported as a common association of left AOUPA^14^ as in our case (Table 4).

UPAA is a rare malformation that can present as an isolated lesion or may be associated with other congenital heart defects.^15^

In normal cardiovascular circulation, there must be no direct communication between the aorta and the pulmonary arteries. A number of congenital anomalies can result in this abnormal pathway, *e.g.* PDA, aorto-pulmonary septal defect and MAPCAs. Patent ductus arteriosus is a vascular structure that connects the proximal descending aorta to the roof of the main pulmonary artery near the origin of the left branch of pulmonary artery.^16^ This essential foetal structure should normally close spontaneously after birth and if persists after the first few weeks of life, it is considered abnormal. The overall incidence of PDA is estimated as 1/500.^16^ PDA can be an isolated cardiovascular defect or it can occur in association with other CHDs. Sometimes, the patency of the ductus arteriosus is critical for the survival in certain CHDs like aortic stenosis and COA in order to maintain the systemic perfusion.^17^

Anomalous pulmonary venous return results in an extracardiac left-to-right shunt, as pulmonary venous blood flows directly into the right side of the heart or into the systemic veins. TAPVC may rapidly result in death without any right to left shunt typically an ASD or a patent foramen ovale.

All of our TAPVC cases were associated with right to left shunt (ASD and VSDs) (Table 6).

LPSVC is the most common congenital anomaly of thoracic venous system with prevalence of 0.3–0.5% in general population. Its diagnosis is usually made as an incidental finding during cardiovascular imaging or surgery (Table 7). Almost 40% of patients with PLSVC can have a variety of associated cardiac anomalies.^18^

### Conclusions

CHDs seem to be a reasonably prevalent health problem in Afghan paediatric population and MDCT angiography can be considered as a non-invasive, available diagnosis tool in evaluation of complex intra- and extracardiac anomalies after initial echocardiography. In Afghanistan, MDCT evaluation of CHD as an alternative becomes more important in a country where there is severe shortage of interventional cardiologists.

### Limitations

This is a retrospective non-ECG gated CT report-based study, therefore no information was collected about coronary artery anomalies. Other possible limitations include lack of breath hold compliance in children. Also, this study cannot be considered as screening study, as patient referred from cardiologist had some degree of symptoms, hence cannot be considered a healthy cohort.

Lastly, as this study was based on imaging performed till 2014, future researchers interested in researching on similar theme are advised to follow Society of Cardiovascular Computed Tomography expert consensus document^19^ in relation to paediatric imaging that was published in 2015.
